# DUSP5P1 promotes gastric cancer metastasis and platinum drug resistance

**DOI:** 10.1038/s41389-022-00441-3

**Published:** 2022-10-28

**Authors:** Xiaohong Wang, Lianhai Zhang, Qiaoyi Liang, Chi Chun Wong, Huarong Chen, Hongyan Gou, Yujuan Dong, Weixin Liu, Ziyu Li, Jiafu Ji, Jun Yu

**Affiliations:** 1 Key laboratory of Carcinogenesis and Translational Research, Peking University Cancer Hospital and Institute, Beijing, China; 2 Institute of Digestive Disease and Department of Medicine and Therapeutics, State Key Laboratory of Digestive Disease, Li Ka Shing Institute of Health Sciences, CUHK-Shenzhen Research Institute, The Chinese University of Hong Kong, Hong Kong, Hong Kong, SAR of China

**Keywords:** Cancer genetics, Gastric cancer, Cancer therapeutic resistance

## Abstract

We elucidated the functional significance and molecular mechanisms of DUSP5P1 lncRNA (dual specificity phosphatase 5 pseudogene 1) in gastric carcinogenesis. We demonstrated that gastric cancer (GC) patients with high DUSP5P1 expression had shortened survival in two independent cohorts. DUSP5P1 promoted GC cell migration and invasion in vitro and metastasis in vivo. Mechanistically, DUSP5P1 activated ARHGAP5 transcription by directly binding to the promoter of ARHGAP5 with a binding motif of TATGTG. RNA-seq revealed that ARHGAP5 activated focal adhesion and MAPK signaling pathways to promote GC metastasis. DUSP5P1 also dysregulated platinum drug resistance pathway. Consistently, DUSP5P1 overexpression in GC cells antagonized cytotoxic effect of Oxaliplatin, and shDUSP5P1 plus Oxaliplatin exerted synergistic effect on inhibiting GC metastasis in vitro and in vivo. DUSP5P1 depletion also suppressed the growth of platinum drug-resistant PDO models. In conclusion, DUSP5P1 promoted GC metastasis by directly modulating ARHGAP5 expression to activate focal adhesion and MAPK pathways, serves as therapeutic target for platinum drug resistant GC, and is an independent prognostic factor in GC.

## Introduction

Gastric cancer (GC) is the third leading cause of cancer-related mortality globally [1, 2]. The proportion of GC patients that present with metastases has increased to over 40%. Metastatic gastric cancer has a poor prognosis. Cytotoxic chemotherapy and radiation therapy remain the backbone of systemic treatment for advanced GC [3]. However, the therapeutic advances have been limited and advanced disease remains incurable. Identification of new molecular factor that mediates GC metastasis may provide new insights into the targeted therapy for advanced GC.

Long noncoding RNAs (lncRNAs) are emerging as a central player in controlling diverse cellular mechanisms. Aberrant expression of lncRNA may have repercussions for cell proliferation, tumor progression, invasion and metastasis [4, 5]. lncRNA may act as enhancers, endogenous siRNA, scaffolds or decoys by physically interacting with other RNA species or proteins, resulting in a direct impact on cell signaling cascades. lncRNAs also have diagnostic and prognostic potential, or even inform therapeutic options for cancer patients. In addition, application of lncRNAs as potential targets for therapeutics holds great promise for future cancer therapy [6, 7].

We reported that C8orf76 promotes GC tumorigenesis through directly binding to the promoter of lncRNA dual-specificity phosphatase 5 pseudogene 1 (DUSP5P1) to activate MAPK signaling [8]. DUSP5P1 is located on chromosome 1q42. DUSP5P1 is highly expressed in Hodgkin’s lymphoma cell lines and several tumor types, but not in normal blood cells [9]. High expression of DUSP5P1 is associated with poor prognosis in acute myeloid leukemia [10]. However, the functional significance and mechanism of DUSP5P1 in GC are largely unknown. In this study, we investigated the functional role, molecular mechanism, and clinical implication of DUSP5P1 in gastric carcinogenesis.

## Materials and methods

### Patients and human samples

Two independent cohorts of patients with histologically confirmed GC were included in the study. Cohort I included 112 paired GC tumor tissues and adjacent non-tumor tissues from Prince of Wales Hospital at the Chinese University of Hong Kong, Hong Kong, and Cohort II included 106 paired GC tumor tissues and adjacent non-tumor tissues from Peking University Cancer Hospital, Beijing, China. The detailed patient information is shown in Table S1. In addition, 23 paired paraffin-embedded primary gastric tumor and adjacent non-tumor tissues, 15 paired paraffin-embedded primary gastric tumor and metastatic lymph node lesion from GC patients were obtained from Peking University Cancer Hospital. TNM stage of GC was classified according to the 7th edition of classification recommended by the American Joint Committee on Cancer (AJCC) [11]. All subjects provided informed consent for obtaining the tissue specimens. This study was approved by the Clinical Research Ethics Committee of the Chinese University of Hong Kong and the Institutional Review Boards of Peking University Cancer Hospital, respectively. This study was carried out in accordance with the Declaration of Helsinki of the World Medical Association.

### Cell lines

Eight GC cell lines (AGS, SGC7901, BGC823, HGC27, MGC803, NCI-N87, MKN45, and MKN74), normal gastric epithelial cell line GES1 and normal liver cell line LO2 were used in this study. AGS, NCI-N87, and LO2 were obtained from ATCC (American Type Culture Collection, Manassas, VA). BGC823, MGC803, SGC7901, and GES1 were obtained from Cell Research Institute (Shanghai, China). MKN74 and MKN45 were obtained from JCRB (Japanese Collection of Research Bioresources Cell Bank, Japan). HGC27 was obtained from RIKEN BRC Cell Bank (Saitama, Japan). All cell lines have been obtained since 2014 and grown fewer than 20 passages. Cell authentication was confirmed by short tandem repeat profiling. Cells were cultured in Dulbecco’s modified Eagle’s medium (Gibco BRL, NewYork, USA) supplemented with 10% fetal bovine serum (Gibco BRL). Routine Mycoplasma testing was performed by PCR.

### In vivo tumorigenicity and metastasis assays

To assess the effect of DUSP5P1 on tumor metastasis, BGC823 cells (1 × 10^6^ cells) stably transfected with DUSP5P1 expression vector or its control vector, MKN45 cells (2 × 10^7^ cells) stably transfected with DUSP5P1 sh-negative control (shNC) or shDUSP5P1 were injected into the tail vein (*n* = 10 per group) or abdominal cavity (*n* = 5 per group) of 6-week-old male Balb/c nude mice, respectively. Four weeks after injection, the mice were sacrificed and examined. The lungs or the celiac implantation tumor including the intestinal tract were dissected and paraffin-embedded. The sections were stained with hematoxylin and eosin (H&E). Metastatic tumors in the lungs or the celiac implantation tumor were counted in a blinded manner.

To establish the liver metastasis mouse model, MKN45 cells (2 × 10^7^ cells) stably transfected with DUSP5P1 shNC or shDUSP5P1 were injected into the spleen of 6-week-old male Balb/c nude mice (*n* = 5 per group). Four weeks after injection, the mice were sacrificed and examined. Livers were dissected and paraffin embedded, and the sections were stained with haematoxylin and eosin (H&E). Metastatic tumors in the livers were counted in a blinded manner. All animal experimental procedures were approved by the Animal Ethics Committee of the Chinese University of Hong Kong.

### Stellaris RNA FISH

Expression of DUSP5P1 in GC was detected using Stellaris RNA FISH by 26 independently probes (Biosearch Technologies, UK) using TMA and cell slides according to the protocol provided by the manufacturer. Probe sequences are listed in Table S2. Briefly, TMA slides and cell slide were fixed in 4% paraformaldehyde and incubated with Proteinase-K for 30 min at 37 °C (cell slides ignored the Proteinase-K step). The slides were hybridized with DUSP5P1 probes (200 nM) for 16 h at 37 °C. Chromosomes were stained with DAPI in PBS for 5 min. The immunofluorescence images were taken with a fluorescence microscope (Olympus, Tokyo, Japan). Two pathologists evaluated the RNA FISH scores in a blinded manner. The intensity of DUSP5P1 staining was scored on a scale of 1–4 as follows: 1 (0–25%, no staining), 2 (26–50%, weak staining), 3 (51–75%, moderate staining) and 4 (76–100%, strong staining). Tissues with scores of 3 and 4 were defined as high expression group, and those with scores of 1 and 2 were classified as low expression.

### Chromatin Isolation by RNA Purification (ChIRP)-sequencing

ChIRP assays were performed using Magna ChIRP^TM^ RNA Interactome Kits (Merck Millipore, Co., USA) [12]. Briefly, 2 × 10^7^ cells were prepared, crosslinked with 1% glutaraldehyde PBS, unreacted glutaraldehyde was quenched with glycine, and then lysed with lysis buffer. Sonication was used to break genomic DNA into small DNA fragments. Odd or even probes were added and used to hybridization DNA fragments corresponding to the gene regions for ChIRP reactions. Input was used as a control. The sequence of odd or even probes is listed in Table S3. The DNA fragments bound by DUSP5P1 were then isolated and sequenced using the Illumina HiSeq X-ten platform (Shanghai Biotechnology Co., China). Sequencing raw reads were pre-processed by filtering out sequencing adapters, short-fragment reads, and other low-quality reads. Bowtie (version 0.12.8) was then used to map the clean reads to the human hg19 reference genome. Peak detection was performed by MACS (https://pypi.python.org/pypi/MACS/1.4.2) as compared with input. The lncRNA-binding motifs were identified using MEME software (http://meme-suite.org/).

### Patient-derived organoid (PDO) model

PDO models were established as previously described [4]. Briefly, the tissues from primary GC patients were chopped into pieces and digested. In this study, we established platinum drug resistant PDO models in vitro by 3–6 months of continuous drug treatment. Then the models were tested by palatium for 5 days. Dissociated cells were then seeded with complete human organoid medium. The cells were passed on to the 6 wells plate and transfected with DUSP5P1 siRNA1, DUSP5P1 siRNA2 or siNC (100 nM). After 6 h, organoids were seeded in 96-well cell culture plates. MALME3 CTG Assay was performed to measure the value in cell-based system using plate reader. Moreover, PDO models were induced to become platinum resistant. This study was approved by the Institutional Review Boards of Peking University Cancer Hospital.

### Statistical analysis

The results were expressed as mean ± SD. Statistical analysis was performed using the Statistical Package for the Social Sciences (SPSS) (standard V.16.0) (IBM Corporation, New York, USA). Crude relative risks (RRs) of death associated with DUSP5P1 expression and other predictor variables were estimated by univariate Cox proportional hazards regression model. Multivariate Cox model was constructed to estimate the adjusted RR for DUSP5P1 expression. Kaplan–Meier analysis was used to compare the survival distributions of two groups with log-rank test. Mann–Whitney U test or Student’s t test was performed to compare the variables of two groups. *P* value < 0.05 was taken as statistical significance, and all tests were two-tailed.

Other details and additional experimental procedures are provided in Supporting Materials and Methods.

## Results

### DUSP5P1 is a poor prognostic factor in patients with GC

**Fig. 1:**
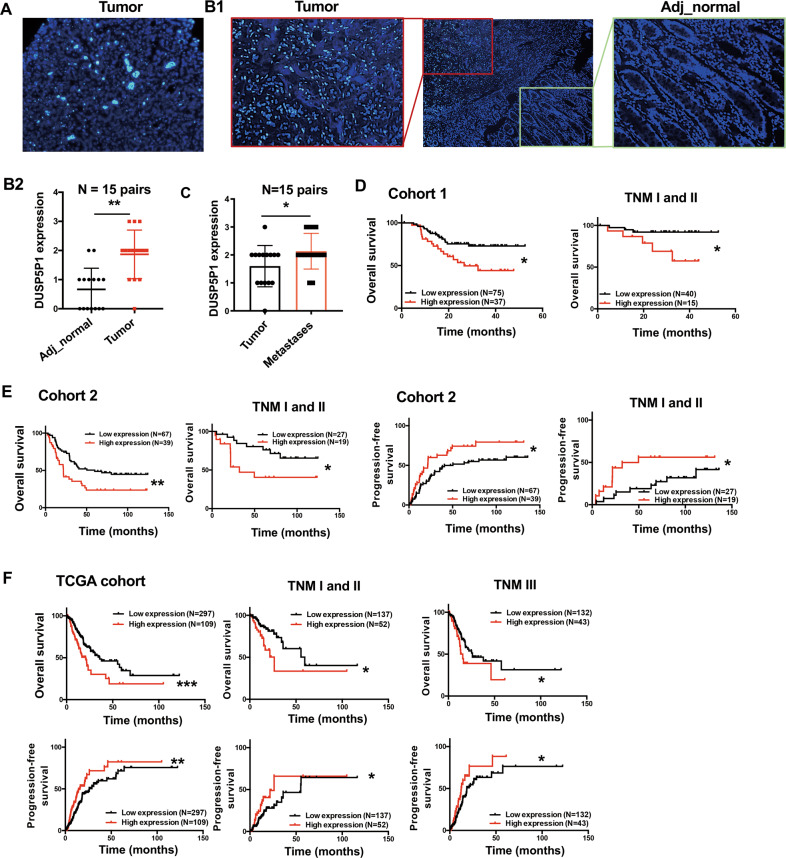
DUSP5P1 upregulation in primary gastric cancers are associated with poor prognosis of gastric cancer patients. **A** Representative images of DUSP5P1 positive staining by RNA FISH in GC tissue. **B** DUSP5P1 expression was significantly upregulated in gastric tumors as compared to adjacent non-tumor tissues by RNA FISH. **C** DUSP5P1 expression was upregulated in metastasis lesions as compared to primary tumor tissues. **D** Kaplan–Meier survival analysis in GC patients with different DUSP5P1 expression status in cohort I. **E** Kaplan–Meier survival analysis in GC patients with different DUSP5P1 expression in cohort II. **F** Kaplan–Meier survival analysis in GC patients with different DUSP5P1 expression in TCGA dataset. *P < 0.05, **P < 0.01, ***P < 0.001, ****P < 0.001.

Representative images of DUSP5P1 expression detected by RNA FISH assay were shown in Fig. 1A. DUSP5P1 expression was significantly increased in primary gastric tumors as compared with adjacent non-tumor tissues (*P* < 0.01, Fig. 1B). DUSP5P1 expression was also significantly higher in metastatic lesions as compared to primary gastric tumors (*P* < 0.05, Fig. 1C). To evaluate the prognostic value of DUSP5P1, its expression was further examined in two cohorts of primary gastric tumor tissues by qRT-PCR (Cohort I: *n* = 112, Cohort II: *n* = 106). The optimal cutoff value of DUSP5P1 expression was determined by ROC curve analysis (*P* = 0.007, Fig. S1). High DUSP5P1 expression was found in 33.04% (37/112) of primary gastric tumors in cohort I, and 36.79% (39/106) in cohort II, respectively.

Kaplan-Meier survival curve indicated that patients with high DUSP5P1 expression had significantly shorter overall survival (OS) than those with low or silenced DUSP5P1 expression in Cohort I (*P* = 0.0118) (Fig. 1D) and Cohort II (*P* = 0.005) (Fig. 1E, left panel). High DUSP5P1 expression predicted a higher risk of cancer-related death by univariate Cox regression analysis (Cohort I: *P* = 0.014; Cohort II: *P* = 0.006) (Table S4). Multivariate Cox regression analysis showed that DUSP5P1 high expression was an independent poor prognostic factor for GC patients (Cohort I: *P* = 0.021; Cohort II: *P* = 0.002) (Table S5). Moreover, DUSP5P1 high expression was also associated with shortened progression-free survival (PFS) (*P* = 0.0154) (Fig. 1E, right panel). After stratification by tumor staging, high DUSP5P1 predicted poor prognosis in stage I-II GC patients both in Cohort I (*P* = 0.0201) (Fig. 1D) and in Cohort II (OS, *P* = 0.0203; PFS, *P* = 0.0482) (Fig. 1E).

Our findings were then validated in TCGA cohort (N = 406). Consistently, Kaplan-Meier survival analysis demonstrated that high DUSP5P1 expression predicted shortened OS and PFS for GC patients (OS: *P* = 0.0005; PFS: *P* = 0.0017) (Fig. 1F), especially for stage I-II GC patients (OS: *P* = 0.0103; PFS: *P* = 0.0391) (Fig. 1F). High DUSP5P1 expression was associated with poor prognosis of GC patients by univariate Cox regression analysis (*P* = 0.001, Table S6) and multivariate Cox regression analysis (*P* = 0.002, Table S7). These results indicated that high DUSP5P1 expression predicted poor prognosis in GC patients at an early stage.

### DUSP5P1 promotes migration and invasion abilities of GC cells

**Fig. 2:**
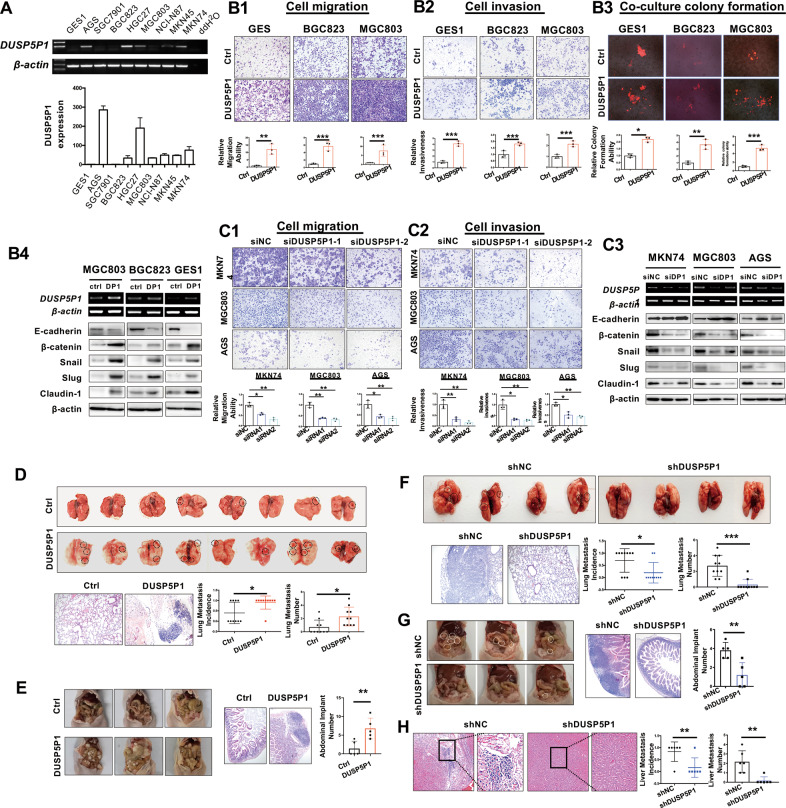
DUSP5P1 promotes GC cell migration, invasion in vitro and metastasis in vivo. **A** DUSP5P1 was expressed in GC cell lines but not in the normal gastric cell line GES-1 by RT-PCR. **B** Representative images of migration and Matrigel invasion transwell assay revealed that ectopic expression of DUSP5P1 promoted cell migration (B1), cell invasion (B2) and RFP-tagged GC cell colonies in co-cultures with LO2(B3) in GES1, BGC823, and MGC803 cells, accompanied by enhanced protein levels of β-catenin, Snail, Slug and Claudin-1, and reduced level of E-cadherin (B4). **C** Representative images of migration and Matrigel invasion transwell assays revealed that knockdown of DUSP5P1 inhibited cell migration (C1) and invasion in MKN74, MGC803 and AGS cells (C2), accompanied by reduced protein levels of β-catenin, Snail, Slug and Claudin-1, and enhanced level of E-cadherin (C3). **D** Representative macroscopic appearance and histological confirmation by HE staining of lung metastasis of GC cells. DUSP5P1 expression in BGC823 cells significantly increased the number of metastatic lesions in the lungs. **E** Representative macroscopic appearances of peritoneal surfaces implanting and HE stained images injected with BGC823 cells transfected DUSP5P1 and control vector were shown. **F** Representative macroscopic appearances of lung metastasis and HE stained images of the lungs injected with MKN45 cells transfected shDUSP5P1 and shNC were shown. **G** Representative macroscopic appearances of peritoneal surfaces implanting and HE stained images injected with MKN45 cells transfected shDUSP5P1 and shNC were shown. **H** Representative HE stained images of the liver were shown. Silencing of DUSP5P1 in MKN45 cells significantly reduced the number of metastatic lesions in the liver. *P < 0.05, **P < 0.01, ***P < 0.001, ****P < 0.001.

DUSP5P1 was highly expressed in 5 out of 8 GC cell lines (AGS, HGC27, MKN74, NCI-N87, and MKN45), but was silenced in the normal gastric epithelial cell line GES1 (Fig. 2A). Ectopic expression of DUSP5P1 in GES1, BGC823, and MGC803 cells promoted cell migration (Fig. 2B1) and invasion (Fig. 2B2). Moreover, we further co-cultured GES1, BGC823, and MGC803 cells expressing control vector or DUSP5P1 together with red fluorescence protein (RFP) with normal liver cells LO2, respectively. Ectopic expression of DUSP5P1 in GES1, BGC823, and MGC803 cells led to RFP + colonies significantly larger in diameter as compared with control cells (Fig. 2B3). Conversely, DUSP5P1 knockdown in MKN74, MGC803, and AGS cells suppressed cell migration and invasion (Fig. 2C1 and C2). These phenotypic effects were confirmed by Western blot showing that DUSP5P1 enhanced the protein expression of epithelial-mesenchymal transition (EMT) markers β-catenin, Snail, Slug, and Claudin-1, whilst expression of E-cadherin was reduced (Fig. 2B4). In contrast, silencing of DUSP5P1 mediated opposite effects on these EMT markers (Fig. 2C3). These results suggested that DUSP5P1 played an important role in promoting pro-metastatic properties of GC cells.

### DUSP5P1 promotes GC cells metastasis to the peritoneum, lung, and liver in vivo

To investigate the tumorigenic ability of DUSP5P1 in vivo, we injected BGC823 cells with stably expressing DUSP5P1 or control vector into the tail vein of nude mice. After four weeks, histological examinations demonstrated that confirmed that 90% (9/10) of the mice bearing BGC823-DUSP5P1 produced lung metastases, while only 40% (4/10) of the mice in BGC823-control vector cells exhibited lung metastases (*P* < 0.05) (Figs. 2D, S2). Moreover, the number of metastatic lesions was significantly higher in DUSP5P1-overexpressing group than in control vector group (*P* < 0.05) (Fig. 2D). We further assessed the effect of DUSP5P1 on peritoneum metastasis in vivo through abdominal implantation. Consistently, more tumor nodules were observed in the peritoneum of the DUSP5P1 group as compared to control group (Figs. 2E, S3).

To confirm the effect of DUSP5P1 on metastasis, we evaluated the metastatic capacity of MKN45 cells with or without DUSP5P1 knockdown in three experimental metastasis models using tail vein (lung), abdominal (peritoneum) or intrasplenic (liver) injection, respectively. At the end of experiment, fewer tumor nodules were observed on the lung (Figs. 2F, S4) and peritoneal surfaces (Figs. 2G, S5) in shDUSP5P1 group as compared to shNC group. Histological examination showed that the average number of metastatic lesions in the lung, liver and peritoneum from shDUSP5P1 group was significantly decreased as compared with shNC group (Fig. 2F–H). Collectively, these results indicated the important role of DUSP5P1 in promoting GC metastasis.

### DUSP5P1 induces focal adhesion and MAPK signaling cascades

**Fig. 3:**
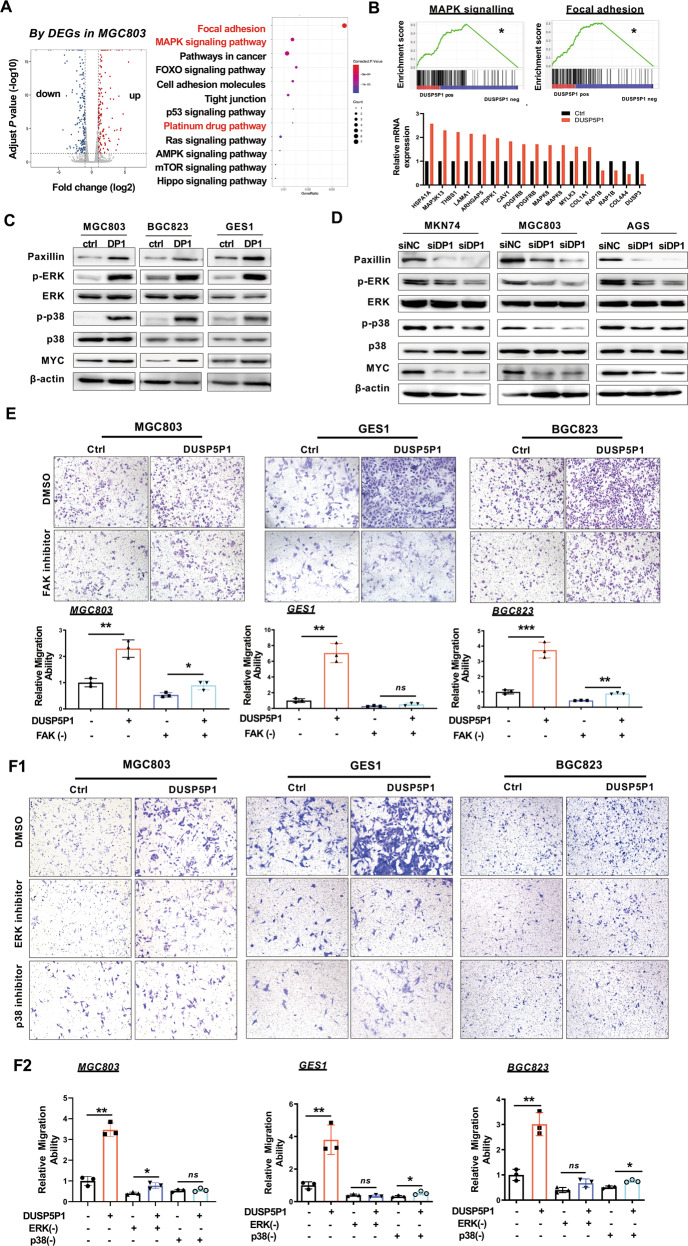
DUSP5P1 activates focal adhesion and MAPK signaling pathway. **A** KEGG pathways enriched by differentially expressed genes affected by DUSP5P1. **B** Enriched differentially expressed genes of focal adhesion and MAPK signaling pathway. **C** DUSP5P1 re-overexpression promoted focal adhesion and MAPK pathway as evidenced by protein expression of factors in MAPK signaling pathway in AGS and GES1 cells by Western blot. **D** DUSP5P1 knockdown inhibited the MAPK pathway as evidenced by decreased protein expression of focal and MAPK signaling pathway. **E** Effect of DUSP5P1 on gastric cell migration in the presence or absence of FAK inhibitor (VS6063) in GES1, MGC803, and BGC823 cells (20μmol/L). **F** Effect of DUSP5P1 on gastric cell migration in the presence or absence of MAPK inhibitor. Effect of DUSP5P1 on gastric cell migration in the presence or absence of ERK inhibitor (PD98059) in GES1 (100 μmol/L), MGC803 (150 μmol/L) and BGC823 (150 μmol/L); Effect of DUSP5P1 on gastric cell migration in the presence or absence of p38 inhibitor (BIRB796) in GES1, MGC803, and BGC823 (100 μmol/L). *P < 0.05, **P < 0.01, ***P < 0.001, ****P < 0.001.

To understand the molecular mechanisms of DUSP5P1 in promotion of GC metastasis, global gene transcriptional profiling was analyzed by RNA-sequencing in DUSP5P1-ectopic expressing MGC803 cells and their control counterparts. Our result showed that ectopic expression of DUSP5P1 significantly dysregulated twelve signaling pathways including focal adhesion, MAPK signaling, pathway in cancer, FOXO signaling, Cell adhesion molecules, tight junction, p53 signaling, platinum drug resistance, Ras signaling, AMPK signaling, mTOR signaling and Hippo signaling pathways. Among them, focal adhesion and MAPK signaling were the most enriched pathways (Fig. 3A). The major differentially expressed genes were MAPK8, ARHGAP5, PDPK1, MAPK14, PDGFRB, LAMA1, CAV1, THBS1, MYLK3, COL1A1, COL4A4, PAP1B (focal adhesion) and MAP3K13, MAPK8, PDGFRB, HSPA1A, DUSP3, RAP1B (MAPK signaling) (Fig. 3B). Consistently, DUSP5P1 increased protein expression of key regulators of focal adhesion and MAPK signaling pathways, including Paxillin, FAK, p-ERK1/2, p-p38, and MYC in MGC803, BGC823 and GES1 cells (Fig. 3C). On the contrary, DUSP5P1 knockdown significantly inhibited protein expression of these factors in MGC803, MKN74, and AGS cells (Fig. 3D). We then tested whether the inhibition of focal adhesion or MAPK pathways could blunt the tumor-promoting effect of DUSP5P1. As shown in Fig. 3E, treatment with focal adhesion inhibitor (FAK inhibitor) abolished the promoting effect of DUSP5P1 on cell migration in BGC823, MGC803, and GES1 cells. Consistent results were obtained using MAPK pathway inhibitors targeting p38 and ERK (Figs. 3F1 and F2). Hence, DUSP5P1 exerts the tumor-promoting effect by activating focal adhesion and MAPK signaling.

### ARHGAP5 is a direct downstream target of DUSP5P1

**Fig. 4:**
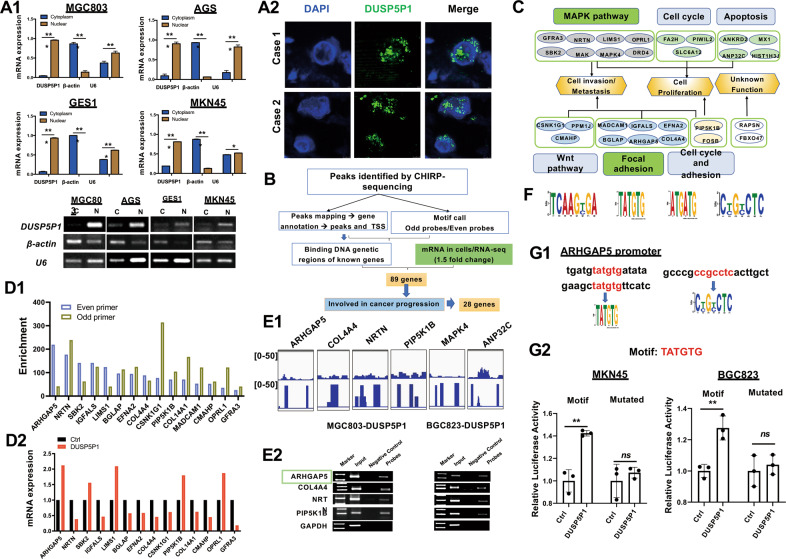
ARHGAP5 is the direct downstream target of DUSP5P1. **A** Analysis of DUSP5P1 location. A1: DUSP5P1 was mainly located in the nucleus of GC cells by RT-PCR after nuclear and cytoplasmic fractionation. A2: Representative images of nuclear localization of DUSP5P1 in GC tissues by RNA FISH. **A** Nuclear localization of DUSP5P1 in DUSP5P1 overexpressed BGC823, GES1 and MGC803 cells following DUSP5P1 transfection by RNA FISH. **B** Flow chart of ChIRP-sequencing assay and RNA-sequencing data analysis of binding site for DUSP5P1 to select the direct downstream target genes of DUSP5P1. **C** The function and pathway enrichment of DUSP5P1 regulated target genes. **D** Downstream targets of DUSP5P1 identified by ChIRP-sequencing and RNA-sequencing. D1: The enrichment fold change of even and odd probes by ChIRP-sequencing and D2: the corresponding change of mRNA expression, respectively. **E** Validation of DUSP5P1 and binding target region of the downstream genes. E1: Representative visual images of enrichment feature of the target regions; E2: ChIRP-PCR was performed to determine the interaction between DUSP5P1 and target region of the downstream genes. **F** Motif analysis of target sequence by ChIRP-sequencing. **G** Analysis of motif ARHGAP5 binded. G1: The binding region and the motif analysis of the ARHGAP5 gene. G2: Luciferase reporter assay showed that DUSP5P1 binds to the “TATGTG” motif. *P < 0.05, **P < 0.01, ***P < 0.001, ****P < 0.001.

We next analyzed the cellular localization of DUSP5P1 by RNA FISH after nuclear and cytoplasmic fractionation. Twenty-six probes of DUSP5P1 were designed for RNA FISH (Table S2). RT-PCR after RNA nuclear and cytoplasmic fractionation and RNA FISH results showed that DUSP5P1 is mainly located in the nucleus of GC cells (Fig. 4A1 and A2). To identify the genomic interactions of DUSP5P1 with its downstream effectors, ten probes complementary to the lncRNA transcript used to purify chromatin bound DUSP5P1 were designed and divided into odd and even groups. ChIRP-seq was then performed in MGC803 cells transfected with DUSP5P1 vector. Eighty-nine DUSP5P1-binding DNA candidates were identified in both odd and even probes group (Fig. 4B). The dysregulated genes were mainly enriched in cell proliferation, cell cycle, cell apoptosis, cell invasion, which involved in MAPK, WNT and focal adhesion pathways (Fig. 4C, D). Among the candidate genes, eighty-nine genes were found to be regulated by DUSP5P1 in RNA-seq analysis (Fig. 4D). Representative visual images of the enrichment feature of the target regions are shown in Fig. 4E1. Conventional ChIRP-PCR assay confirmed that DUSP5P1 bound to DNA motifs within ARHGAP5, COL4A4, NRTN, and PIP5K1B genes (randomly selected region of GAPDH as control) (Fig. 4E2), indicating that DUSP5P1 may regulate transcription of these candidate genes. Based on the results of both RNA-seq and KEGG pathway analysis, ARHGAP5 were chosen for further investigation. Motif analysis was conducted using the MEME online motif comment tool to discover binding motifs. Typical NFAT ‘core’ binding motifs (and their reversed strands) were identified in odd or even probes group, respectively. Among them, four motifs were identified, including TCAAGT/CGA, TATGTG, ATGATG, and CT/CGT/CCTC (Fig. 4F). DUSP5P1-binding sites in ARHGAP5 DNA sequence were mapped to the promoter region with two binding motifs (TATGTG, CT/CGT/CCTC) (Fig. 4G1). To further validate the regulatory effect of DUSP5P1, we cloned these binding motifs into the promoter region of the pGL3 reporter for luciferase assay. As shown in Fig. 4G2, DUSP5P1 significantly altered the activity of TATGTG motif in MKN45 and BGC823 cells, while motif deletion significantly diminished DUSP5P1-mediated the activity of TATGTG as evidenced by luciferase reporter assay. These results demonstrated that DUSP5P1 directly binds to the promoter of ARHGAP5 and upregulates its transcription.

### Pro-metastatic function of DUSP5P1 is dependent on upregulation of ARHGAP5

**Fig. 5:**
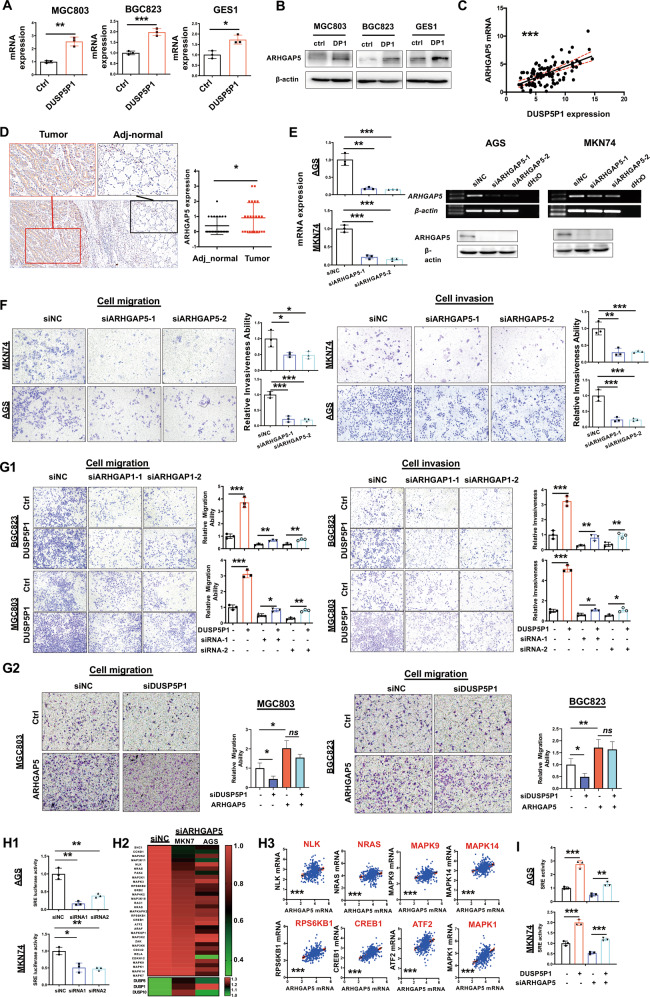
DUSP5P1 exerts tumor-promoting function partially depending on the ARHGAP5. **A**, **B** ARHGAP5 mRNA **A** and protein expression **B** were correlated with the DUSP5P1 expression. Ectopic expression of DUSP5P1 increased the mRNA and protein expression of ARHGAP5 in BGC823, MGC803 and GES1 by qRT-PCR and Western blot. **C** DUSP5P1 expression positively correlated with ARHGAP5. **D** Representative images of ARHGAP5 protein expression in primary GC tissues by IHC (left panel). ARHGAP5 expression significantly higher in the GC tissue as compared with adjacent non-cancer tissues (right panel). **E** Successful knockdown of ARHGAP5 in AGS and MKN74 cells was confirmed by RT-PCR and Western blot. **F** ARHGAP5 knockdown inhibited cell migration and invasion. **G** Pro-metastatic function of DUSP5P1 is dependent on ARHGAP5. (G1) Knockdown of ARHGAP5 significantly blunted the promoting effects of DUSP5P1 on cell migration and invasion. (G2) Effect of DUSP5P1 knockdown on migration ability with or without ARHGAP5 overexpression was investigated. ARHGAP5 overexpression significantly blunted the inhibiting effects of DUSP5P1 knockdown on cell migration. **H** ARHGAP5 activated MAPK signaling. H1: ARHGAP5 activated MAPK signaling as evidenced by SRE luciferase reporter assay (left panel); H2: The effectors of MAPK signaling pathway regulated by ARHGAP5 identified by MAPK signaling pathway PCR array; H3: Correlation analysis of ARHGAP5 and effector genes form TCGA database. **I** Effect of DUSP5P1 on MAPK pathway with or without ARHGAP5 knockdown was detected by SRE luciferase reporter assay. *P < 0.05, **P < 0.01, ***P < 0.001, ****P < 0.001.

Ectopic expression of DUSP5P1 significantly upregulated transcription and increased the expression of ARHGAP5 in BGC823, MGC803, and GES1 cells (Fig. 5A, B). Moreover, DUSP5P1 expression was positively correlated with the mRNA expression of ARHGAP5 (*P* < 0.001, Fig. 5C). ARHGAP5 protein expression was significantly higher in primary gastric tumors as compared with adjacent non-tumor tissues by immunohistochemical (IHC) staining (*P* < 0.05, Fig. 5D). Depletion of ARHGAP5 in MKN74 and AGS cells with normal endogenous DUSP5P1 expression (Fig. 5E) significantly decreased cell migration and invasion (Fig. 5F). We then assessed whether the pro-metastatic function of DUSP5P1 in GC was dependent on ARHGAP5. ARHGAP5 knockdown in DUSP5P1-overexpressing BGC823 and MGC803 cells significantly blunted the promoting effects of DUSP5P1 on cell migration and invasion (Fig. 5G1), while ARHGAP5 overexpression significantly diminished DUSP5P1 KO-mediated inhibition of migration in MGC803 and BGC823 cells (Fig. 5G2), indicating that the tumor-promoting effect of DUSP5P1 is at least in part dependent on ARHGAP5 in GC.

Given that focal adhesion promotes MAPK pathway, the effect of ARHGAP5 on MAPK pathway was investigated by SRE luciferase reporter assay. ARHGAP5 depletion reduced MAPK activities in both AGS and MKN74 cells (Fig. 5H1). The effects of ARHGAP5 on MAPK signaling components are investigated using MAPK pathway PCR array by qRT-PCR (Fig. 5H2). The major differentially expressed genes such as NLK, NRAS, MAPK9, MAPK14, RPS6KB1, CREB1, ATF2, and MAPK1 were further confirmed by TCGA dataset (Fig. 5H3). Moreover, knockdown of ARHGAP5 significantly diminished DUSP5P1-mediated MAPK signaling activation as evidenced by luciferase reporter assay (Fig. 5I).

### DUSP5P1 is a potential therapeutic target

**Fig. 6:**
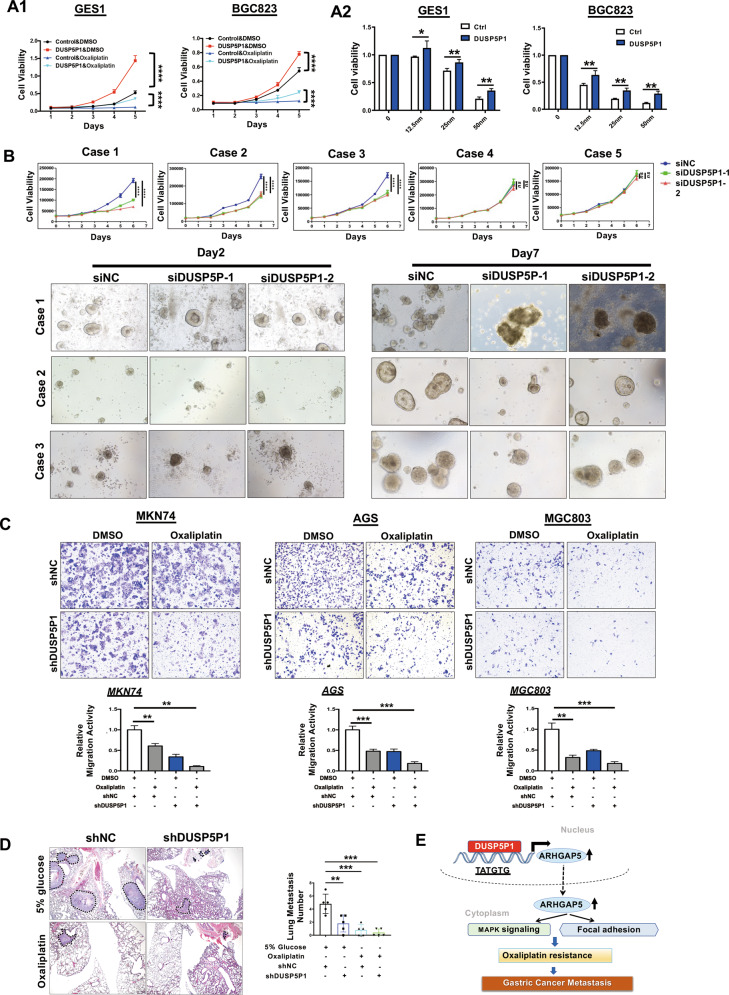
DUSP5P1 is a potential therapy target in platinum drugs resistant GC cells. **A** DUSP5P1 transfected GC cells was partially resistant to the chemotherapy effect of Oxaliplatin by MTT assay. **B** DUSP5P1 Knockdown suppressed the GC PDO cell growth in the three of five platinum drugs resistant models. **C** Combination therapy of Oxaliplatin and DUSP5P1 depletion significantly inhibited the migration of the GC cells. **D** Representative HE staining image of the lung metastasis revealed metastasis synergistic inhibiting effect of Oxaliplatin and DUSP5P1 depletion in vivo. **E** Proposed mechanistic scheme of DUSP5P1 promoting the GC progression. DUSP5P1 promoted the expression of ARHGAP5, by directly binding to promoter region, which further promotes the activity of focal adhesion and MAPK signaling pathway. *P < 0.05, **P < 0.01, ***P < 0.001, ****P < 0.001.

Previous reports indicated that focal adhesion molecules, MAPK signaling pathway and specific lncRNA in tumor cells induced resistance to chemotherapy [13, 14]. Our results also showed that DUSP5P1 significantly dysregulated platinum drug resistance pathway (Fig. 3A). We thus asked whether DUSP5P1 affects chemotherapeutic efficacy in GC cells. GES1 and BGC823 cells stably transfected with DUSP5P1 were treated with Oxaliplatin (5μmol for 6 days). We found that DUSP5P1 overexpression partially counteracted the cytotoxic effect of Oxaliplatin in both GES1 and AGS cells (Fig. 6A1). Moreover, DUSP5P1 ectopic expressing GC cells also demonstrated Oxaliplatin resistance over a range of drug dosages (Fig. 6A2). To test if DUSP5P1 is a potential therapeutic target for platinum drug-resistant GC, we established five personalized platinum drugs resistant PDO models generated from advanced GC cases with different clinicopathological features (Table S8). As shown in Fig. 6B, knockdown of DUSP5P1 significantly suppressed the GC cell growth in the three of five platinum drugs resistant PDO models (Fig. S6). Moreover, ARHGAP5 overexpression significantly diminished DUSP5P1 KO-mediated inhibition of cell growth after Oxaliplatin treated (Fig. S7). Our findings suggested that DUSP5P1 may serve as a potential target in platinum drugs resistant GC patients.

The synergistic effect of the combination of DUSP5P1 depletion and Oxaliplatin on cell migration was then investigated. MKN74, MGC803 and AGS cells with stable knockdown of DUSP5P1 were treated with Oxaliplatin at 25 μmol for 36 h. Combined Oxaliplatin treatment and shDUSP5P1 showed a synergistic effect on suppressing the cell migration as compared with DUSP5P1 depletion alone (Fig. 6C). To confirm the synergistic effect of DUSP5P1 on metastasis in vivo, we inoculated nude mice with MKN45 cells administration stably transfected with shDUSP5P1 or shNC via tail vein, and portal vein, and treated with 5 mg/kg Oxaliplatin (i.p.) or 5% glucose (i.p., vehicle), respectively. Combination of Oxaliplatin and shDUSP5P1 showed a synergistic effect on suppressing lung metastasis as compared with DUSP5P1 depletion or Oxaliplatin treatment alone (Figs. 6D, S8). Collectively, these results demonstrated the synergistic effect of DUSP5P1 depletion and Oxaliplatin on inhibiting GC metastasis.

## Discussion

In this study, we found that DUSP5P1 is highly expressed in GC tumor tissues. High expression of DUSP5P1 is associated with poor prognosis of GC patients independent of their clinical pathological features, especially for early stage GC patients, implying that DUSP5P1 is an independent prognostic factor of GC patients.

A series of bio-functional experiments revealed that DUSP5P1 significantly promoted cell migration/invasion abilities in vitro, and lung, liver, and peritoneal metastasis of GC cells in nude mice in vivo; whilst depletion of DUSP5P1 by shRNA in MKN45 cells inhibited lung and liver metastasis in vivo. These results collectively confirmed the pro-metastatic effect of DUSP5P1 in GC. Concomitantly with the promotion of metastases, we demonstrated that DUSP5P1 facilitated EMT transition through the upregulation of mesenchymal critical regulators (β-catenin, Snail, Slug and Claudin-1) [15] and down-regulation of epithelial marker E-cadherin [16].

The molecular mechanisms by which DUSP5P1 exerts its pro-tumorigenesis and pro-metastases functions in GC were then investigated. We demonstrated that DUSP5P1 caused significant dysregulation of signaling pathways, in particular, focal adhesion and MAPK signaling, which are the key regulating pathways of cancer progression and metastasis [13, 17]. We confirmed DUSP5P1 significantly induced activation of focal adhesion and MAPK, as evidenced by the enhanced protein expression of key factors in these two signaling pathways including Paxillin, FAK, p-ERK1/2, p-p38, and MYC. Inhibiting focal adhesion and MAPK signaling by specific inhibitors significantly blunted the pro-metastatic effect of DUSP5P1 in GC cells. Thus, DUSP5P1 plays an important tumor promoting role in gastric metastasis through dysregulated oncogenic signaling pathways, especially focal adhesion and MAPK signaling.

As DUSP5P1 was localized primarily to the nucleus, we hypothesized that DUSP5P1 may regulate transcription in GC. We performed integrated ChIRP-sequencing [18] and RNA-sequencing analyses, which revealed that ARHGAP5 is a critical downstream target of DUSP5P1. From ChIRP-PCR assay, we revealed that DUSP5P1 dysregulates the transcription of alternative downstream factors ARHGAP5, COL4A4, NRTN, and PIP5K1B, which might also contribute to the function of DUSP5P1. The direct binding motif of DUSP5P1 to the promoter region of ARHGAP5 is identified (TATGTG) and confirmed by ChIRP-PCR. DUSP5P1 actively induced transcription activity and expression of ARHGAP5. ARHGAP5 is functionally involved in DUSP5P1-mediated GC metastasis. We found that ARHGAP5 significantly promoted GC metastasis by inducing cell migration and invasion. Particularly, ARHGAP5 knockdown by siRNA significantly abolished the cell migration and invasion promoting effect mediated by DUSP5P1. Hence, ARHGAP5 is the direct downstream effector of DUSP5P1 and the pro-metastatic effect of DUSP5P1 is partially dependent on ARHGAP5 in GC. Based on data from rescue assays using ARHGAP5 overexpression in DUSP5P1-silenced cells, we estimated that approximately 50% of DUSP5P1 activity is dependent on ARHGAP5. ARHGAP5 function is intrinsically linked to RhoA regulation [19]. In agreement with our data, ARHGAP5 was shown to induce metastatic dissemination in different cancers [20, 21]. Moreover, ARHGAP5 is a key regulator of the focal adhesion signaling pathway [22], which functions as major regulators of MAPK activities [23]. Consistently, we showed that ARHGAP5 knockdown in GC cells significantly inhibited MAPK signaling pathway. ARHGAP5 knockdown also significantly blunted DUSP5P1-induced activation of MAPK signaling, suggesting that ARHGAP5 plays a pivotal role in DUSP5P1-mediated activation of MAPK signaling in GC.

Emerging reports indicate that lncRNAs predict responsiveness to chemotherapy and may function as potential drug targets [14]. We therefore examined the possibility that DUSP5P1 could affect chemotherapy in GC cells. Indeed, DUSP5P1 overexpression reduced the chemotherapy efficacy of Oxaliplatin in GC cells. Moreover, in our inhouse platinum drugs resistant GC PDOs, knockdown of DUSP5P1 significantly suppressed cell growth in three out of five platinum drugs resistant PDO models. Combination of Oxaliplatin with shDUSP5P1 synergistically suppressed cell migration of GC cells in vitro as well as lung and liver metastasis in vivo as compared with DUSP5P1 depletion or Oxaliplatin treatment alone, implying that DUSP5P1 serves as a potential target in platinum drug-resistant GC patients.

In conclusion, our study reported for the first time that lncRNA DUSP5P1 is a novel tumor metastasis-promoting factor in GC. The molecular mechanisms of DUSP5P1 as a tumor-promoting factor involved direct induction of the tumor promoting factor ARHGAP5, resulting in the activation of focal adhesion and MAPK signaling pathways (Fig. 6E). DUSP5P1 might serve as a novel prognostic biomarker and therapeutic target for metastatic GC patients.

## Supplementary information

Supplemental material and methods

Supplemental figures

Supplemental tables

## Data Availability

All the ChiRP and RNA-sequencing data presented in the current study are publicly available in the GEO database (accession number: GSE213582).

## References

[1] Bray F, Ferlay J, Soerjomataram I, Siegel RL, Torre LA, Jemal A (2018). Global cancer statistics 2018: GLOBOCAN estimates of incidence and mortality worldwide for 36 cancers in 185 countries. CA Cancer J Clin.

[2] Yang L, Zheng R, Wang N, Yuan Y, Liu S, Li H (2018). Incidence and mortality of stomach cancer in China, 2014. Chin J Cancer Res.

[3] Thomassen I, van Gestel YR, van Ramshorst B, Luyer MD, Bosscha K, Nienhuijs SW (2014). Peritoneal carcinomatosis of gastric origin: a population-based study on incidence, survival and risk factors. Int J Cancer.

[4] Flockhart RJ, Webster DE, Qu K, Mascarenhas N, Kovalski J, Kretz M (2012). BRAFV600E remodels the melanocyte transcriptome and induces BANCR to regulate melanoma cell migration. Genome Res.

[5] Huarte M (2015). The emerging role of lncRNAs in cancer. Nat Med.

[6] Wang KC, Chang HY (2011). Molecular mechanisms of long noncoding RNAs. Mol Cell.

[7] Wang KC, Yang YW, Liu B, Sanyal A, Corces-Zimmerman R, Chen Y (2011). A long noncoding RNA maintains active chromatin to coordinate homeotic gene expression. Nature.

[8] Wang X, Liang Q, Zhang L, Gou H, Li Z, Chen H (2019). C8orf76 Promotes Gastric Tumorigenicity and Metastasis by Directly Inducing lncRNA DUSP5P1 and Associates with Patient Outcomes. Clin Cancer Res.

[9] Staege MS, Muller K, Kewitz S, Volkmer I, Mauz-Korholz C, Bernig T (2014). Expression of dual-specificity phosphatase 5 pseudogene 1 (DUSP5P1) in tumor cells. PLoS One.

[10] Zhou LY, Yin JY, Tang Q, Zhai LL, Zhang TJ, Wang YX (2015). High expression of dual-specificity phosphatase 5 pseudogene 1 (DUSP5P1) is associated with poor prognosis in acute myeloid leukemia. Int J Clin Exp Pathol.

[11] Washington K (2010). 7th edition of the AJCC cancer staging manual: stomach. Ann Surg Oncol.

[12] Chu C, Chang HY (2016). Understanding RNA-chromatin interactions using chromatin isolation by RNA purification (ChIRP). Methods Mol Biol.

[13] Eke I, Cordes N (2015). Focal adhesion signaling and therapy resistance in cancer. Semin Cancer Biol.

[14] Schmitt AM, Chang HY (2016). Long noncoding RNAs in cancer pathways. Cancer Cell.

[15] Bronsert P, Enderle-Ammour K, Bader M, Timme S, Kuehs M, Csanadi A (2014). Cancer cell invasion and EMT marker expression: a three-dimensional study of the human cancer-host interface. J Pathol.

[16] Canel M, Serrels A, Frame MC, Brunton VG (2013). E-cadherin-integrin crosstalk in cancer invasion and metastasis. J Cell Sci.

[17] Mitsuno Y, Yoshida H, Maeda S, Ogura K, Hirata Y, Kawabe T (2001). Helicobacter pylori induced transactivation of SRE and AP-1 through the ERK signalling pathway in gastric cancer cells. Gut.

[18] Chu C, Qu K, Zhong FL, Artandi SE, Chang HY (2011). Genomic maps of long noncoding RNA occupancy reveal principles of RNA-chromatin interactions. Mol Cell.

[19] Stiegler AL, Boggon TJ (2017). p190RhoGAP proteins contain pseudoGTPase domains. Nat Commun.

[20] Fang Y, Zhu X, Wang J, Li N, Li D, Sakib N (2015). MiR-744 functions as a proto-oncogene in nasopharyngeal carcinoma progression and metastasis via transcriptional control of ARHGAP5. Oncotarget.

[21] Wang J, Tian X, Han R, Zhang X, Wang X, Shen H (2014). Downregulation of miR-486-5p contributes to tumor progression and metastasis by targeting protumorigenic ARHGAP5 in lung cancer. Oncogene.

[22] Zrihan-Licht S, Fu Y, Settleman J, Schinkmann K, Shaw L, Keydar I (2000). RAFTK/Pyk2 tyrosine kinase mediates the association of p190 RhoGAP with RasGAP and is involved in breast cancer cell invasion. Oncogene.

[23] Zhang H, Shao H, Golubovskaya VM, Chen H, Cance W, Adjei AA (2016). Efficacy of focal adhesion kinase inhibition in non-small cell lung cancer with oncogenically activated MAPK pathways. Br J Cancer.

