# Supplementary material for: DUSP5P1 promotes gastric cancer metastasis and platinum drug resistance
Source: Oncogenesis. 2022 Oct 28;11(1):66. doi: 10.1038/s41389-022-00441-3 (PMC9616843; doi:10.1038/s41389-022-00441-3)
Supplement: Supplementary file 1 — Supplemental material and methods [file 41389_2022_441_MOESM1_ESM.docx]

**Supplementary MATERIALS AND METHODS**

**DUSP5P1 promotes gastric cancer metastasis and platinum drug resistance**

**Short title**: DUSP5P1 sensitizes gastric adenocarcinoma to metastasis

**RNA extraction and real-time PCR analyses**

Total RNA was extracted using TRIzol and transcribed into cDNA using a High Capacity cDNA Kit (Applied Biosystems, Foster City, CA). For quantitative PCR analysis, aliquots of cDNA were amplified using SYBR® Premix Ex TaqTM II (Takara Bio Inc, Japan) on LightCycler® 480 Instrument (Roche Diagnostics, Switzerland). Each sample was tested in triplicate. ∆∆CT method was employed to determine the fold change in gene expression level. ΔCT method was employed to determine the relative expression levels of corresponding genes. The sequences of primers used are listed in **Table S9**.

**Western blot**

Protein lysates from cell lines and tissues were prepared using protease inhibitor cocktail (Roche)–containing radioimmunoprecipitation assay (RIPA) buffer. Protein concentration was determined by the DC protein assay method of Bradford (Bio-Rad, Hercules, CA). Proteins were separated on sodium dodecyl sulfate-polyacrylamide gel electrophoresis (SDS-PAGE) and transferred onto nitrocellulose membranes (GE Healthcare, Piscataway, NJ). Blots were immunostained with primary antibodies at 4°C overnight and secondary antibody at room temperature for 1 hour. Proteins were visualized using ECL Plus Western Blotting Detection Reagents (GE Healthcare). The antibodies used in this study are listed in **Table S10**.

**Immunohistochemistry**

Immunohistochemistry for ARHGAP5 was performed on paraffin sections using anti-ARHGAP5 antibody, respectively (**Table S10**). The extent of staining was assessed by the areas of focal positivity of cytoplasm staining: 0, <5% was defined as low expression, 1+, 6-25%, 2+, 26-50%; 3+, 51-100% were defined as high expression. IHC score was independently evaluated by two blinded scorers.

**Construction of gene expression plasmid and establishment of stable DUSP5P1 expressing cells**

The full-length open reading frame sequence of DUSP5P1 was obtained by RT-PCR amplification of normal human gastric cDNA. The PCR aliquots were subcloned into the pcDNA3.1 vector and then verified by DNA sequencing. Either DUSP5P1 or control vector was transfected into GC cells using lipofectamine 2000 (Life Technologies, Carlsbad, CA). Overexpression of DUSP5P1 in GES1, MGC803 or BGC823 cells were selected with G418 antibiotics for 2 weeks.

**Lenti-virus-mediated shRNA targeting DUSP5P1 knockdown and ARHGAP5 transient knockdown**

Knock-down DUSP5P1 expression in AGS, MGC803 and MKN74 cell lines was performed by a lenti-virus-mediated shRNA targeting DUSP5P1. Both shNegative control (NC) and shDUSP5P1 (5’ - GGAGGTGCCTACCATGATCC - 3’; 5’ - GCATGATCTCACCCAGCTTTG - 3’) (Shanghai Genepharma Co., Ltd, China) cells were selected with puromycin for 2 weeks after transfection for 48h. For ARHGAP5 knockdown, AGS and MKN74 cells were transfected with 50nM ARHGAP5 NM_001030055 siRNA (5’- GCA CUG AUC AGC UAG GCU UTT- 3’; 5’ – CCG GGU GCC UUU GGC ACA UTT - 3’) (Shanghai Genepharma Co., Ltd, China) or control siRNA (Shanghai Genepharma Co., Ltd, China) using lipofectamine 2000 (Life Technologies).

**Invasion assays**

For invasion assays, matrigel-coated chambers (Becton Dickinson, Waltham, MA) were used. Briefly, 1×10^4^ cells were seeded into the upper chamber in serum-free culture medium. The lower chamber was filled with completed medium with 10% FBS. After 48h, cells that have invaded through the membrane were stained with crystal violet and counted.

**Dual-luciferase reporter assay**

SRE (Serum Response Element) signaling pathway luciferase reporters were examined. The cell lines in 24-well plates and were co-transfected with luciferase report plasmid (0.2 μg/well) and pRL-cyto-megalovirus (pCMV) vector (5ng/well) using lipofectamine 2000 (Life Technologies). Cells were harvested 48hours post-transfection and luciferase activities were analyzed by the dual-luciferase reporter assay system (Promega).

**RNA-sequencing**

The total amount of 3 μg RNA per sample was used as input material for the RNA sample preparations. All samples had RIN values above 6.8. Sequencing libraries were generated using IlluminaTruSeqTM RNA Sample Preparation Kit (Illumina, San Diego, CA), following manufacturer’s recommendations. The libraries were sequenced on an Illumina HiSeq X-ten platform as per manufacturer’s instructions (Shanghai Biotechnology Co., China).

**Patient derived Organoid (PDO) model**

Surgery tissues were obtained from patients. The tissues were washed with cold PBS containing antibiotics and chopped into approximately 5-mm pieces with surgical scissors. Tissues were further washed with 10 mL Advanced DMEM/F12 (Thermo Fisher Scientific, Waltham, MA) containing 1x Glutamax, 10mMHEPES, and antibiotics and digested in 10 mL Advanced DMEM/F12 containing 2% FCS and 2 mg/ml collagenase (Sigma, C9407) on an orbital shaker at 37℃ for 1-2h. The pellet was resuspended in 10ml Advanced DMEM/F12 containing 2% FCS and centrifuged again at 400 rcf. Dissociated cells were collected in Advanced DMEM, suspended in growth factor reduced (GFR) matrigel (Corning Inc., Corning, NY), and seeded. The matrigel was then solidified and overlaid with 500μl of complete human organoid medium, which was subsequently refreshed every two days. PDOs were cultured in Advanced DMEM/F12, supplemented with 1x B27 additive and 1x N2 additive (Thermo Fisher Scientific), 0.01% bovine serum albumine, 2 mM L-glutamine, 100 units/ml penicillin-streptomycin, and containing the following additives: EGF, noggin, R-spondin 1, gastrin, FGF-10, FGFF-basic, Wnt-3A, prostaglandin E2, Y-27632, nicotinamide, A83-01, A83-01, SB202190, HGF (Pepro-Tech, London, UK). Passaging of PDOs was performed using Trypsin. PDOs were biobanked in FBS (Thermo Fisher Scientific), containing 10% DMSO (Sigma- Aldrich, St Louis, MI). The organoid cells were passed onto the 6 wells plate and transfected with C8orf76 siRNA or negative control siRNA (100nM). After 6 hours, organoids were seeded in 96-well cell culture plates; after matrigel solidified it was overlaid with 70 μl of complete human organoid medium. It was conducted in triplicate. The sequence of siNC and siC8orf76 is same with the cell lines which is indicated in the Supporting Materials and methods. MALME3 CTG Assay was performed to measure the value in cell-based system using plate reader every 24 hours.
