# Supplementary material for: DUSP5P1 promotes gastric cancer metastasis and platinum drug resistance
Source: Oncogenesis. 2022 Oct 28;11(1):66. doi: 10.1038/s41389-022-00441-3 (PMC9616843; doi:10.1038/s41389-022-00441-3)

Figure S1. ROC curve analysis of DUSP5P1 expression.

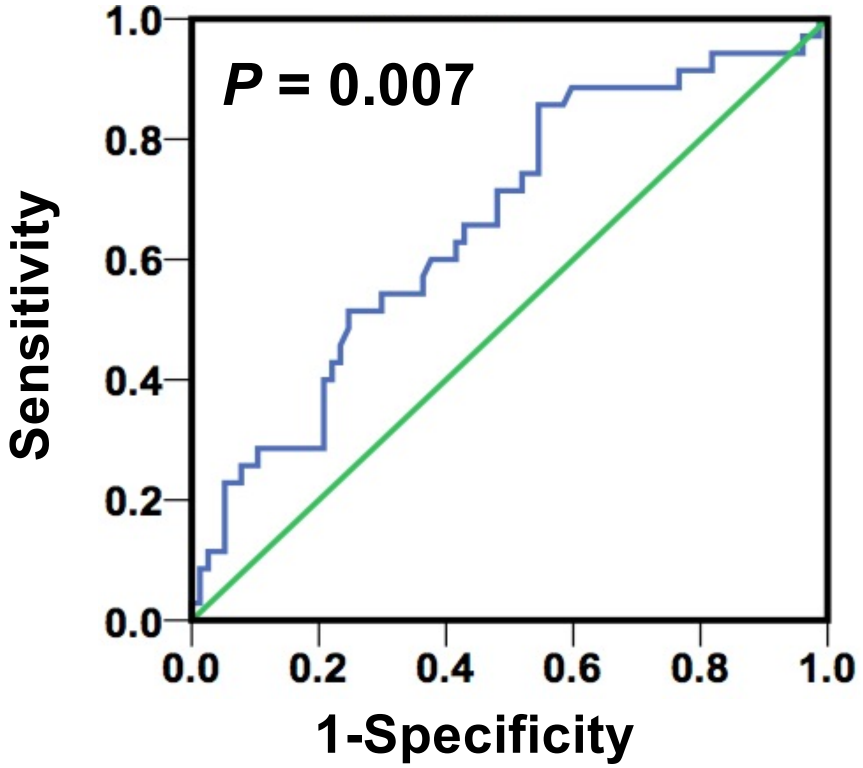

Figure S2. Macroscopic appearance of lung metastasis injected with BGC823 cells transfected DUSP5P1 and control vector of GC cells.

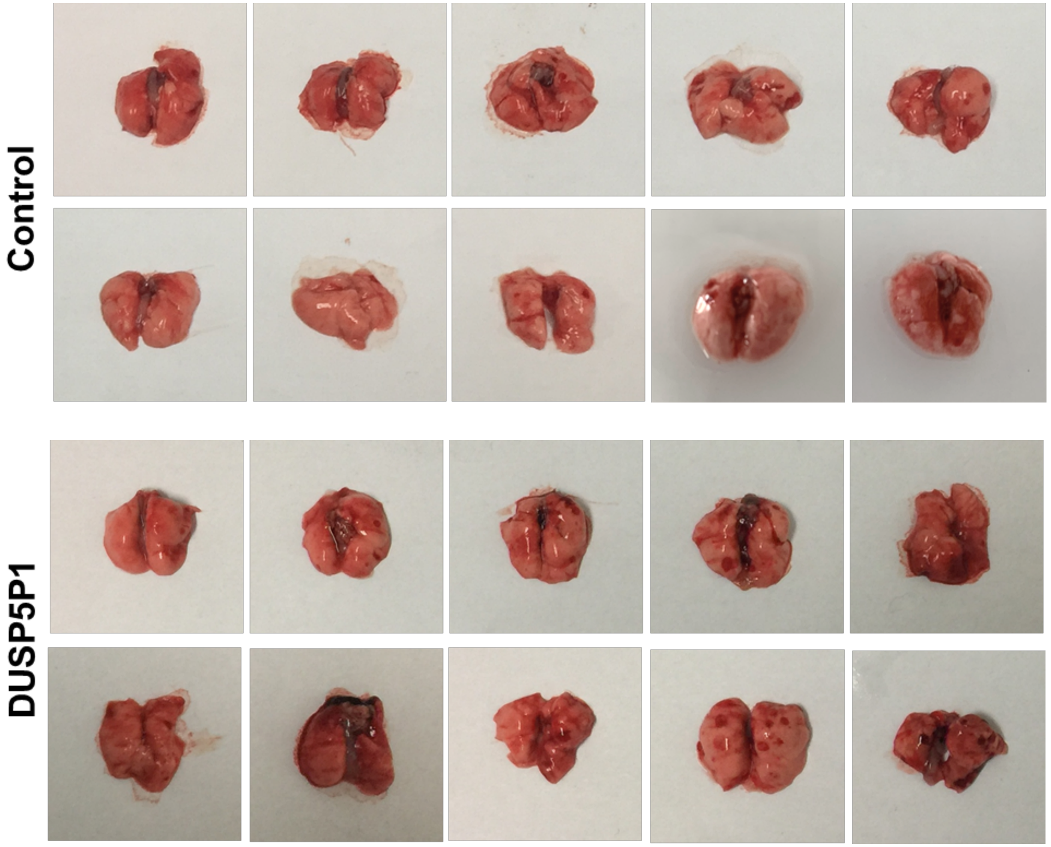

Figure S3. Macroscopic appearances of peritoneal surfaces implanting injected with BGC823 cells transfected DUSP5P1 and control vector

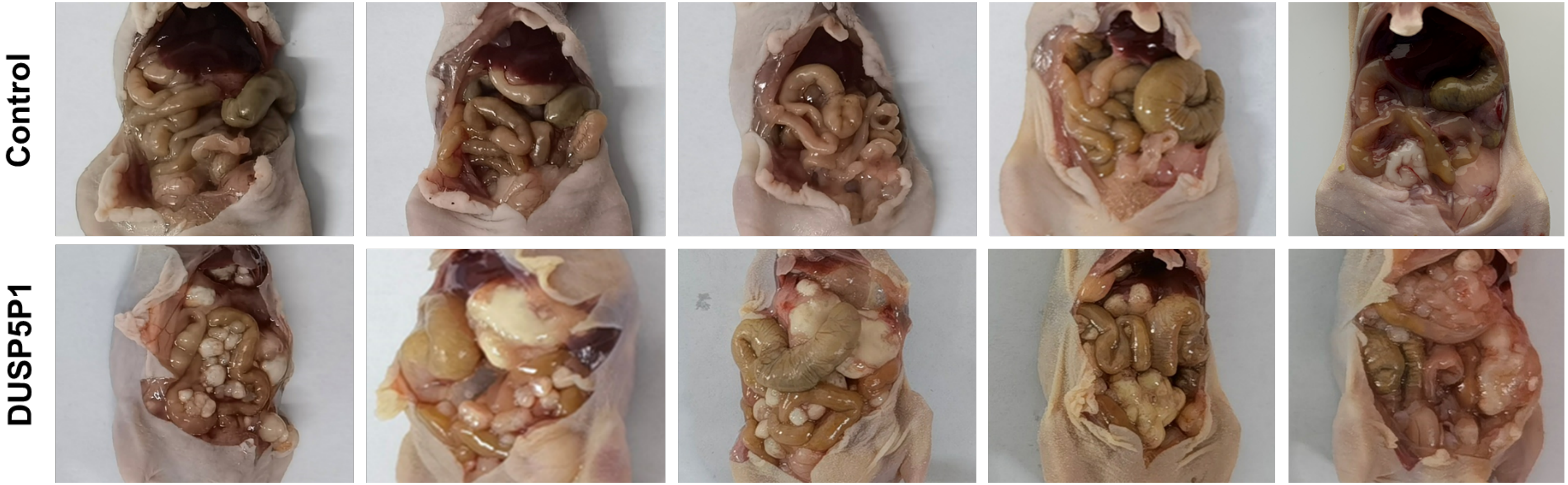

Figure S4. Macroscopic appearances of lung metastasis of the lungs injected with MKN45 cells transfected shDUSP5P1 and shNC.

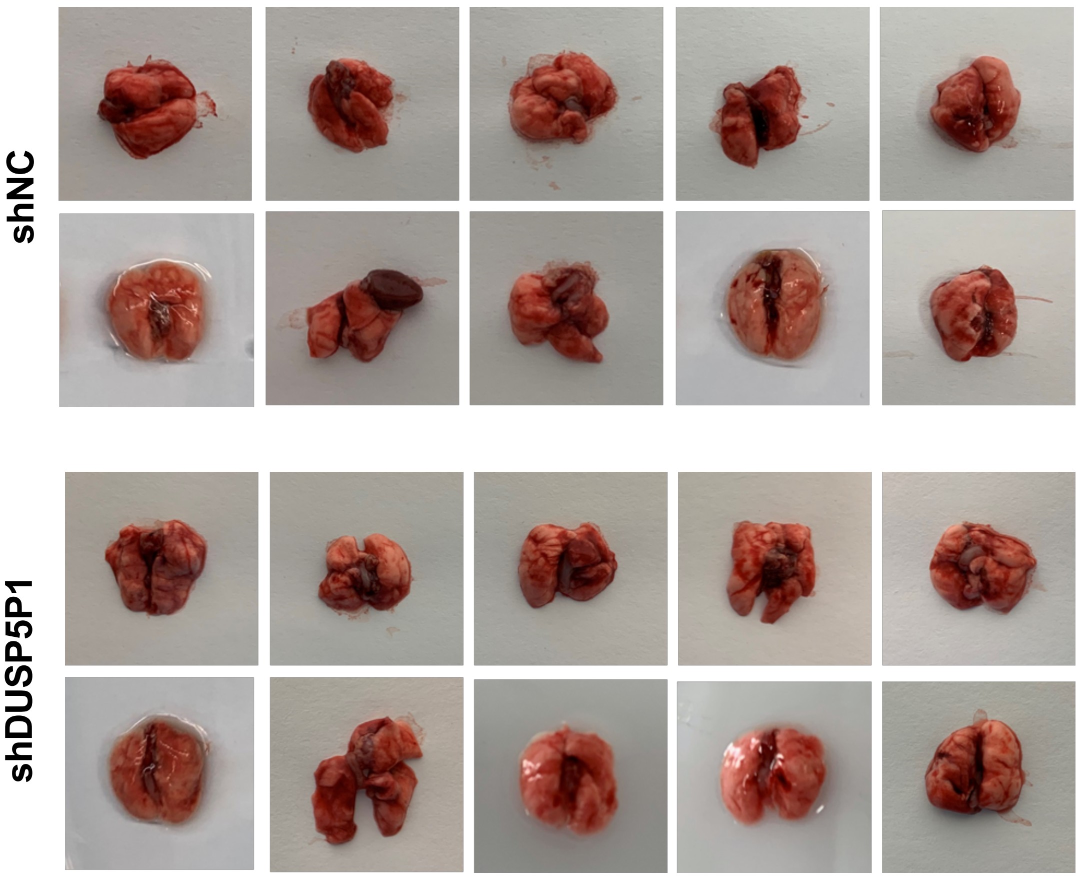

Figure S5. Macroscopic appearances of peritoneal surfaces implanting injected with MKN45 cells transfected shDUSP5P1 and shNC.

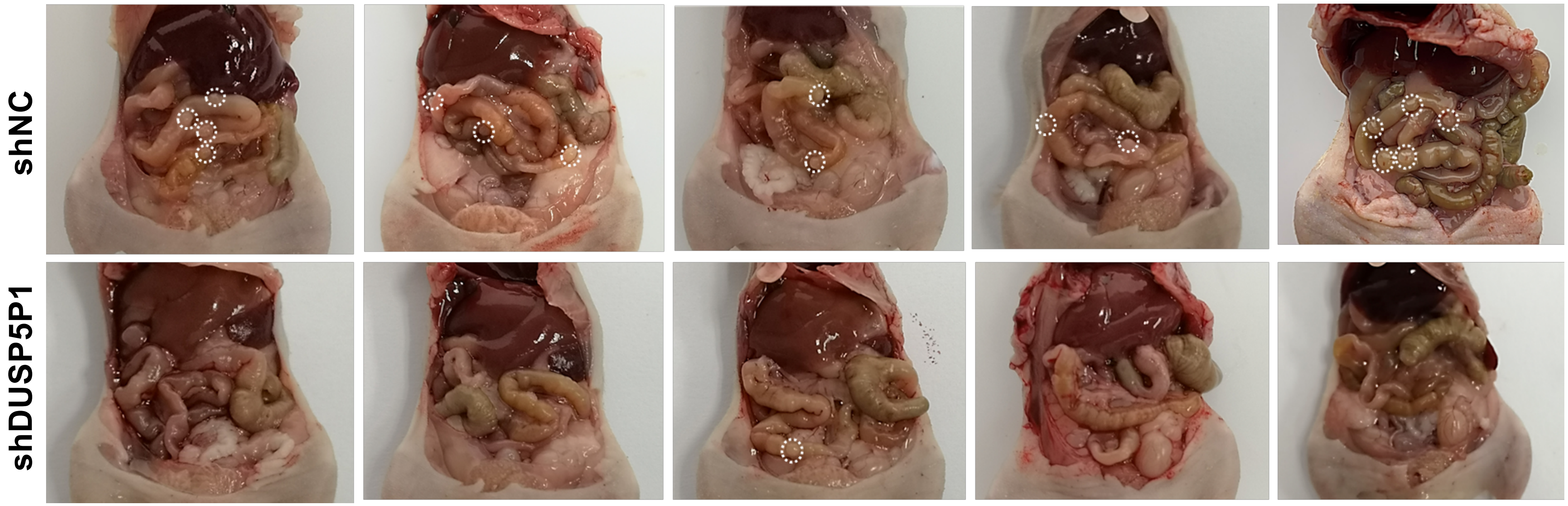

Figure S6. Establishment of the platinum-resistant PDO model.

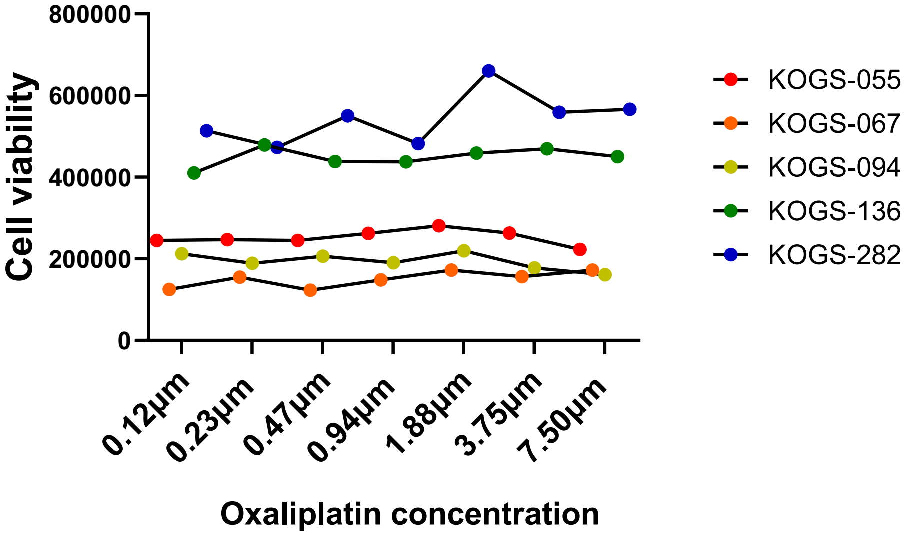

Figure S7. Knockdown of ARHGAP5 blunted the platinum resistant effects of DUSP5P1.

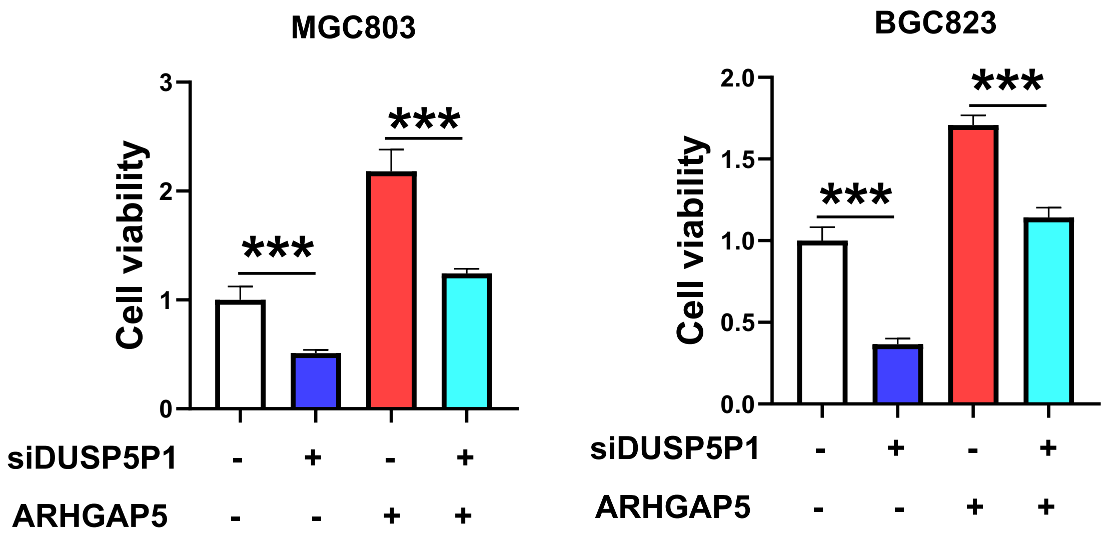

Figure S8. Macroscopic appearances of lung metastasis of the lungs revealed metastasis synergistic inhibiting effect of Oxaliplatin and DUSP5P1 depletion in vivo.

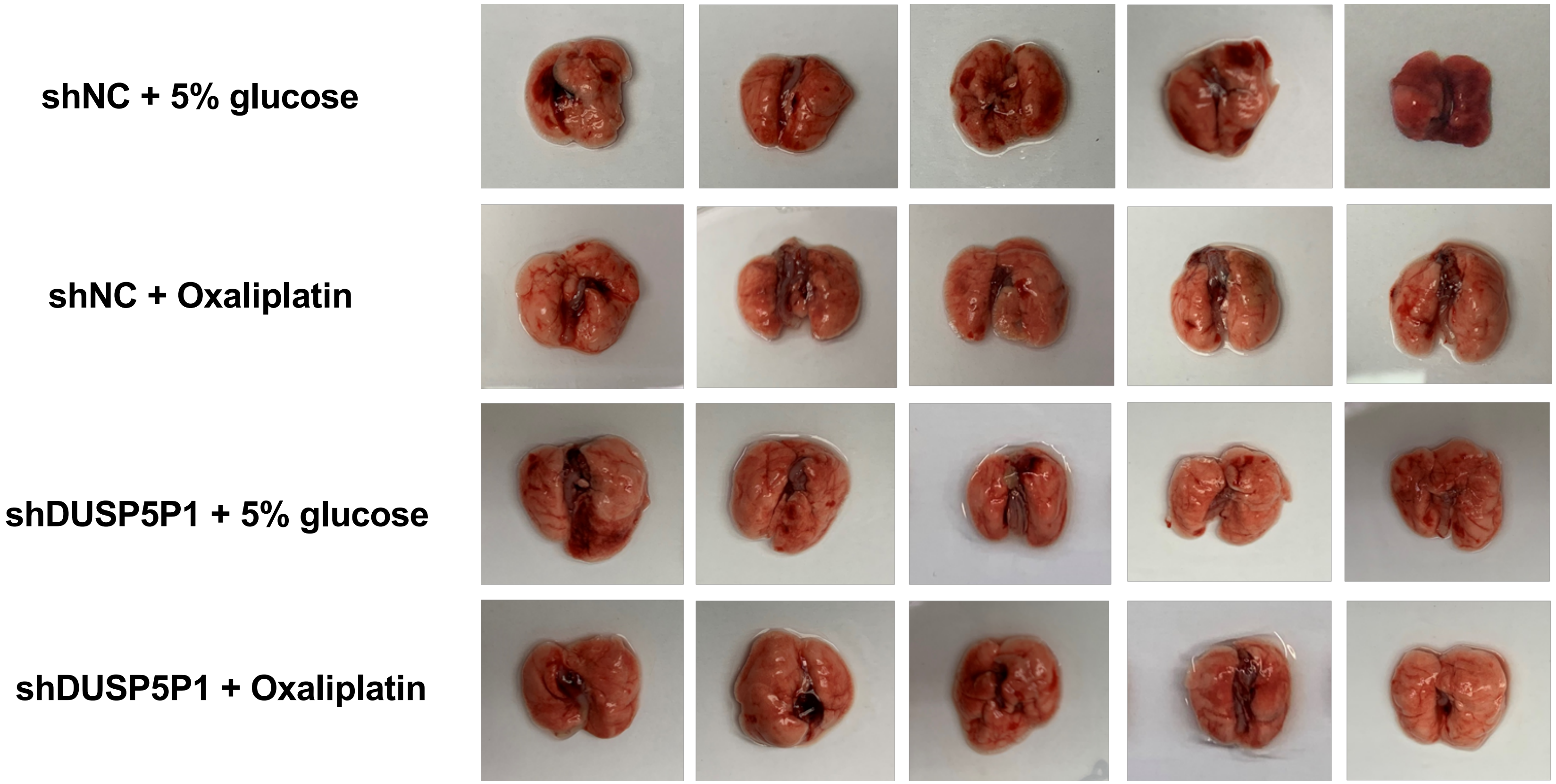

Supplement: Supplementary file 2 — Supplemental figures [file 41389_2022_441_MOESM2_ESM.pdf]
