# Supplementary material for: DUSP5P1 promotes gastric cancer metastasis and platinum drug resistance
Source: Oncogenesis. 2022 Oct 28;11(1):66. doi: 10.1038/s41389-022-00441-3 (PMC9616843; doi:10.1038/s41389-022-00441-3)
Supplement: Supplementary file 3 — Supplemental tables [file 41389_2022_441_MOESM3_ESM.docx]

**Supplementary Tables**

**Table S1.** Clinical and pathological characteristics of patients in two cohorts.

**Table S2.** Stellaris Probe set for DUSP5P1 RNA FISH assay.

**Table S3.** Odd and even probes for the ChIRP-sequencing and ChIRP-PCR assay.

**Table S4.** Univariate cox regression analysis of potential poor prognostic factors for GC patients in two cohorts.

**Table S5.** DUSP5P1 overexpression is an independent poor prognostic factor for GC patients in two cohorts.

**Table S6.** Univariate cox regression analysis of potential poor prognostic factors for GC patients in TCGA datasets.

**Table S7.** DUSP5P1 overexpression is an independent poor prognostic factor for GC patients in TCGA datasets.

**Table S8.** The sample information of PDO models.

**Table S9.** DNA sequences of primers used in this study.

**Table S10.** A list shows the antibodies used.

**Table S1. Clinical and pathological characteristics of patients in two cohorts.**

|  | **Cohort I**  **(Hongkong)** | **Cohort II (Beijing)** |
| --- | --- | --- |
| **No. of patient** | 112 | 106 |
| **Follow-up** (months; mean) | 23.87.34±12.57 | 74.84±48.58 |
| **Age at resection** (years; mean) | 66.45±10.41 | 60.95±10.53 |
| **Gender** |  |  |
| Male | 71 (63.39%) | 77 (72.64%) |
| Female | 41 (36.61%) | 29 (27.36%) |
| **Lauren Subtype** |  |  |
| Intestinal Type | 73 (65.18%) | 45 (42.45%) |
| Diffuse Type | 39 (34.82%) | 61 (57.55%) |
| **TNM Stage** |  |  |
| I+ II | 55 (49.11%) | 46 (44.40%) |
| III+IV | 46 (41.07%) | 60 (56.60%) |
| *Data incomplete in cohort I. |  |  |
| **DUSP5P1** |  |  |
| Low-expression | 75 (66.96%) | 67(63.21%) |
| High-expression | 37 (33.04%) | 39(36.79%) |

**Table S2. Stellaris Probe set for DUSP5P1 RNA FISH assay(5'-3').**

|  | **Stellaris Probes** |
| --- | --- |
| Probe 1 | ttggtcctctcgcacgga |
| Probe 2 | ctaaccgcagtgcagctc |
| Probe 3 | gccgctgatggaactagg |
| Probe 4 | cacgtactccacaggtgc |
| Probe 5 | tcctctatcccagtttta |
| Probe 6 | tagctgctcttggggctg |
| Probe 7 | ggtcttcagtattgcaca |
| Probe 8 | acctctcactatgtcact |
| Probe 9 | gttcatttgcaagcctgg |
| Probe 10 | taccctgaggtctgtgtg |
| Probe 11 | ccttccttcaatcctgag |
| Probe 12 | gtgctcctgtaattgctt |
| Probe 13 | gtacagtagtcagcacgt |
| Probe 14 | ttgagggcagggggtctg |
| Probe 15 | ggcgaactttgaggtgca |
| Probe 16 | gcaggtatttattgcctt |
| Probe 17 | agtgccatttctgttggt |
| Probe 18 | ctccacaaaccctgtagg |
| Probe 19 | tcagggttcaaattggca |
| Probe 20 | ctccattggacccaagag |
| Probe 21 | ggccaacatcttgctact |
| Probe 22 | gtccaaggtcagtgagga |
| Probe 23 | gccaccttgatggtatga |
| Probe 24 | aagtacccttttcctcct |
| Probe 25 | actgacccaggatacaca |
| Probe 26 | gtcagtggcttgaagaca |

**Table S3. Odd and even probes for the ChIRP-sequencing and ChIRP-PCR assay(5'-3').**

|  | **Odd probes** | **Even probes** |
| --- | --- | --- |
| Probe 1 | tgatggaactaggccctaac | gttcctctatcccagtttta |
| Probe 2 | tgacctctcactatgtcact | tgtgctcctgtaattgcttg |
| Probe 3 | gaaaaggcgaactttgaggt | gaattggcttcataagtgcc |
| Probe 4 | ggcatatatatcatgtctcc | aaagtccaaggtcagtgagg |
| Probe 5 | attactgacccaggatacac | aagtcagtggcttgaagaca |

**Table S4. Univariate cox regression analysis of potential poor prognostic factors for GC patients in two cohorts.**

| **Variables** | **Univariate Cox regression analysis** | |
| --- | --- | --- |
|  | **HR（95%CI)** | ***P* value** |
| **Cohort I (HK cohort)** |  |  |
| **Age** | 1.031(0.997-1.066) | 0.073 |
| **Gender** |  |  |
| Male vs. Female | 1.540(0.791-2.997) | 0.204 |
| **DUSP5P1 expression** |  |  |
| High vs. Low | 2.289(1.179-4.443) | 0.014 |
| **Lauren subtype** |  |  |
| Diffuse vs. Intestinal | 1.206(0.613-2.373) | 0.587 |
| **TNM stage** |  |  |
| III and IV vs. I and II | 5.113(2.293-11.401) | <0.001 |
| **Cohort II (BJ cohort)** |  |  |
| **Age** | 1.026(1.000-1.053) | 0.048 |
| **Gender** |  |  |
| Male vs. Female | 1.205(0.674-2.155) | 0.530 |
| **DUSP5P1 expression** |  |  |
| Positive vs. Negative | 2.030(1.230-3.349) | 0.006 |
| **Lauren subtype** |  |  |
| Diffuse vs. Intestinal | 1.189(0.669-2.024) | 0.522 |
| **TNM stage** |  |  |
| III and IV vs. I and II | 2.678(1.545-4.644) | <0.001 |

**Table S5. DUSP5P1 overexpression is an independent poor prognostic factor for GC patients in two cohorts.**

| **Variables** | **Multivariate Cox regression analysis** | |
| --- | --- | --- |
|  | **HR（95%CI)** | ***P* value** |
| **Cohort I (HK cohort）** |  |  |
| **DUSP5P1 expression** |  |  |
| (**High *vs* Low)** | 2.253(1.130-4.493) | 0.021 |
| **TNM stage (III/IV *vs* I/II)** | 4.620(2.064-10.344) | <0.001 |
| **Cohort II（BJ cohort）** |  |  |
| **DUSP5P1 expression** |  |  |
| (**High *vs* Low)** | 2.236(1.352-3.696) | 0.002 |
| **TNM stage (III/IV *vs* I/II)** | 2.876(1.655-4.995) | <0.001 |

**Table S6. Univariate cox regression analysis of potential poor prognostic factors for GC patients in TCGA datasets.**

| **Variables** | **Univariate Cox regression analysis** | |
| --- | --- | --- |
|  | **HR（95%CI)** | ***P* value** |
| **TCGA cohort** |  |  |
| **Age** | 1.020(1.002-1.037) | 0.025 |
| **Gender** |  |  |
| Female vs. Male | 0.735(0.506-1.066) | 0.105 |
| **DUSP5P1 expression** |  |  |
| High vs. Low | 1.859(1.302-2.654) | 0.001 |
| **Location** |  |  |
| Non-Cardia vs. Cardia | 0.886(0.602-1.304) | 0.540 |
| **Histologic grade** |  |  |
| Diffuse vs. Intestinal | 1.402(0.983-1.999) | 0.062 |
| **TNM stage** |  |  |
| III and IV vs. I and II | 1.766(1.233-2.528) | 0.002 |

**Table S7. DUSP5P1 overexpression is an independent poor prognostic factor for GC patients in TCGA datasets.**

| **Variables** | **Multivariate Cox regression analysis** | |
| --- | --- | --- |
|  | **HR（95%CI)** | ***P* value** |
| **TCGA cohort** |  |  |
| **DUSP5P1 expression** |  |  |
| (**high *vs* low)** | 1.792(1.245-2.580) | 0.002 |
| **Age** | 1.025(1.007-1.044) | 0.007 |
| **TNM stage (III/IV *vs* I/II)** | 1.973(1.367-2.847) | <0.0001 |

**Table S8. The sample information of PDO models.**

| **No** | **Gender** | **Age** | **Pathological diagnosis** | **Lauren subtype** | **HER2** | **TNM** |
| --- | --- | --- | --- | --- | --- | --- |
| 1 | Male | 74 | poorly differentiated adenocarcinoma | Diffuse | 1+ | Ⅳ |
| 2 | Male | 64 | Poorly differentiated adenocarcinoma | Intestinal | 1+ | Ⅳ |
| 3 | Male | 62 | Poorly differentiated adenocarcinoma | Intestinal | 2+ | Ⅳ |
| 4 | Male | 56 | Poorly differentiated adenocarcinoma | Mixed | 3+ | Ⅳ |
| 5 | Male | 50 | Moderately to poorly differentiated adenocarcinoma, partly signet ring cell carcinoma | Intestinal | - | Ⅳ |

**Table S9. DNA sequences of primers used in this study.**

| **Primer name** | **Sequence (5'-3')** |
| --- | --- |
| **RT-PCR** |  |
| β-actin-F | CATCCACGAAACTACCTTCAACTCC |
| β-actin-R | GAGCCGCCGATCCACACG |
| DUSP5P1-F | CTGCGGTTAGGGCCTAGTTC |
| DUSP5P1-R | GTACTCCACAGGTGCCCAAG |
| ARHGAP5-F | AGGGAAGCTCAACGTAGATGG |
| ARHGAP5-R | ATGATCCACGCATTCATCACAT |
| COL4A4-F | GAGGGGTCTTTCAGGAGTGC |
| COL4A4-R | GCATGTCCCTCTCTGCCTTT |
| **ChIRP-PCR** |  |
| ARHGAP5-F | CTGAGATTGCGCCACTGC |
| ARHGAP5-R | TCACCGTATCAGCCAGGATA |
| COL4A4-F | GCTGACCTGCCTTCTACCA |
| COL4A4-R | AGAGAGAGAGAGAGAAGGAAGG |
| MADCAM1-F | TGAGGCTCCATGTCCAATTT |
| MADCAM1-R | GTCCCTGAAGGAACCCTTGT |
| NRTN-F | TTGGAGGCTGAGGTGGGTAT |
| NRTN -R | AGTGTAGTGGCGCGATCTC |
| PIP5K1B-F | TTGTCCAGGCCAGTCTTGAA |
| PIP5K1B-R | GAAGGGCCGGGCATGGTA |
| GAPDH-F | TACTAGCGGTTTTACGGGCG |
| GAPDH-R | TCGAACAGGAGGAGCAGAGAGCGA |
| ARHGAP5-target1 | CGGGAGGCTGAGGCCGGGGAATGGC |
| ARHGAP5-target2 | GCCATTCCCCGGCCTCAGCCTCCCG |

**Table S10. A list shows the antibodies used.**

| **Antibody name** | **Company** | **Catalog No.** | **Dilution** |
| --- | --- | --- | --- |
| β-actin | Santa cruz | sc-47778 | 1:1000 (WB) |
| E-cadherin | Cell Signaling Technology | #3195 | 1:1000 (WB) |
| β-catenin | Cell Signaling Technology | #8480 | 1:1000 (WB) |
| Slug | Cell Signaling Technology | #9585 | 1:1000 (WB) |
| Snail | Cell Signaling Technology | #3879 | 1:1000 (WB) |
| Claudin-1 | Cell Signaling Technology | #13255 | 1:1000 (WB) |
| FAK | Cell Signaling Technology | #3285 | 1:1000 (WB) |
| Paxillin | Cell Signaling Technology | #12065 | 1:1000 (WB) |
| phospho-p38 MAPK | Cell Signaling Technology | #4511 | 1:1000 (WB) |
| phospho-ERK1/2 MAPK | Cell Signaling Technology | #4376 | 1:1000 (WB) |
| p38 MAPK | Cell Signaling Technology | #8690 | 1:1000 (WB) |
| ERK1/2 MAPK | Cell Signaling Technology | #4695 | 1:1000 (WB) |
| c-myc | Cell Signaling Technology | #9252 | 1:1000 (WB) |
| ARHGAP5 | Proteintech | #55165-1-AP | 1:1000 (WB) |
| ARHGAP5 | Abcam | Ab199160 | 1:200 (IHC) |
| COL4A4 | LifeSpan BioScience | LS‑C145052 | 1:400 (ICC) |
